# Suramin Inhibits Chikungunya Virus Entry and Transmission

**DOI:** 10.1371/journal.pone.0133511

**Published:** 2015-07-24

**Authors:** Yi-Jung Ho, Yu-Ming Wang, Jeng-wei Lu, Tzong-Yuan Wu, Liang-In Lin, Szu-Cheng Kuo, Chang-Chi Lin

**Affiliations:** 1 Institute of Preventive Medicine, National Defense Medical Center, Taipei, Taiwan; 2 Graduate Institute of Life Sciences, National Defense Medical Center, Taipei, Taiwan; 3 Department and Graduate Institute of Microbiology and Immunology, National Defense Medical Center, Taipei, Taiwan; 4 Department of Pathology, and Graduate Institute of Pathology and Parasitology, National Defense Medical Center, Taipei, Taiwan; 5 Department of Clinical Laboratory Sciences and Medical Biotechnology, National Taiwan University, Taipei, Taiwan; 6 Department of Bioscience Technology, Chung Yuan Christian University, Chung-Li, Taiwan; 7 Department of Laboratory Medicine, National Taiwan University Hospital, Taipei, Taiwan; CEA, FRANCE

## Abstract

The mosquito-borne Chikungunya virus (CHIKV) is a profound global threat due to its high rate of contagion and the lack of vaccine or effective treatment. Suramin is a symmetric polyanionic naphthylurea that is widely used in the clinical treatment of parasite infections. Numerous studies have reported the broad antiviral activities of suramin; however, inhibition effects against CHIKV have not yet been demonstrated. The aim of this study was thus to investigate the antiviral effect of suramin on CHIKV infection and to elucidate the molecular mechanism underlying inhibition using plaque reduction assay, RT-qPCR, western blot analysis, and plaque assay. Microneutralization assay was used to determine the EC_50_ of suramin in the CHIKV-S27 strain as well as in three other clinical strains (0611aTw, 0810bTw and 0706aTw). Time-of-addition was used to reveal the anti-CHIKV mechanism of suramin. We also evaluated anti-CHIKV activity with regard to viral entry, virus release, and cell-to-cell transmission. Cytopathic effect, viral RNA, viral protein, and the virus yield of CHIKV infection were shown to diminish in the presence of suramin in a dose-dependent manner. Suramin was also shown the inhibitory activities of the three clinical isolates. Suramin inhibited the early progression of CHIKV infection, due perhaps to interference with virus fusion and binding, which subsequently prevented viral entry. Results of a molecular docking simulation indicate that suramin may embed within the cavity of the E1/E2 heterodimer to interfere with their function. Suramin was also shown to reduce viral release and cell-to-cell transmission of CHIKV. In conclusion, Suramin shows considerable potential as a novel anti-CHIKV agent targeting viral entry, extracellular transmission, and cell-to-cell transmission.

## Introduction

Chikungunya fever is a mosquito-borne disease which causes fever and strong joint pain in humans. Prior to 2005, only sporadic CHIKV outbreaks had been reported in Africa and South Asia; however, in 2005–2006, a large outbreak with nearly a million suspected cases was reported in the Indian Ocean region [1,2]. Furthermore, CHIKV has been suspected in almost 1 million cases of disease in over 20 Caribbean and Central and South American countries since December 2013 [3–5]. Overall, millions of cases of CHIKV have been reported in over 50 countries.

*Aedes spp*. (particularly *Aedes aegypti* and *Aedes albopictus*) are the major vectors of CHIKV. The viral incubation period is generally 2–12 days with major clinical symptoms that include acute fever, maculopapular rash, headache, vomiting, myalgia, and chronic arthralgia. In addition, many patients suffer severe joint pain from weeks or even months [6,7]. CHIKV has been associated with endothelial, epithelial fibroblast cells [7,8], osteoblasts [9], muscle satellite cells [10], monocytes [11] and macrophages [12]. At present, no vaccines or antiviral drugs have been developed for the treatment of CHIKV infection [13].

CHIKV is a plus sense, single-strand enveloped RNA virus classified into the genus *Alphavirus* of the family *Togaviridae*. The genome of CHIKV is approximately 11.8kb in size, comprising two open reading frames (OFRs), encoded nonstructural proteins (nsPs), and structural proteins (E1, E2, E3, K6 and capsid) [7]. Alphavirus infection is established through receptor-mediated endocytosis. The low-pH environment in the endosome induces an irreversible conformational change in glycoproteins and dissociation of the E2/E1 heterodimers followed by E1 trimerization. This causes the release of viral RNA through the virion-endosome membrane fusion [14]. Following this, the nsPs are translated to form replicase complexes for viral replication. E2 and E1 glycoproteins are initially synthesized in the ER and modified in the Golgi apparatus. CHIKV can be spread through either extracellular transmission or cell-to-cell transmission [15] and glycoproteins also involved in [16–18]. Indeed, glycoproteins play a critical role in both early and late stages of CHIKV infection.

Suramin is a symmetrical hexasulfonated naphthylurea compound, which has obtained U.S. Food and Drug Administration (FDA) approval for human use in the treatment of trypanosomiasis. The anti-neoplastic effects of suramin have been demonstrated, and the broad anti-viral activities of this compound have been studied with regard to human T-cell lymphotropic virus (HTLV-III) [19], HIV-1 [20], HSV-1 [21], HBV [22], HCV [23], dengue virus [24], encephalitis B virus [25], SFTSV [26], norovirus [27], EV71 [28], and Rift Valley Fever Virus [29].

This study demonstrates the inhibitory effects of suramin on CHIKV infection. Our results show that suramin blocks CHIKV fusion to host cells directly, primarily through the regulation of the entry step in the CHIKV life cycle. Suramin was also shown to inhibit both extracellular transmission and cell-to-cell transmission following initial CHIKV infection. These data suggest that understanding the mechanism underlying the anti-viral effects of suramin in CHIKV-human host interactions may facilitate the development of plausible treatments to deal with CHIKV infection.

## Materials and Methods

### Cells, viruses and drugs

BHK-21 (ATCC: CCL-10) and U2OS cells (ATCC: HTB-96) were grown in Dulbecco’s modified Eagle medium (DMEM) (Invitrogen, catalog # 10564–011) supplemented with 5% heat inactivated Fetal bovine serum (FBS) (Invitrogen, catalog # 10082–147) and antibiotics under 5% CO_2_ at 37°C. MRC-5 (ATCC: CCL-171) were grown in Minimum Essential medium (MEM) (Invitrogen, catalog # 11095–080) supplemented with 5% FBS and antibiotics under 5% CO_2_ at 37°C. Sf21 cells were cultured with Sf-900II serum-free medium (Invitrogen, catalog # 10902–096) containing 5% heat inactivated FBS and antibiotics at 27°C. Chikungunya virus (ATCC: VR-64, strain S27-African prototype) (CHIKV-S27) and three clinical isolates, 0611aTw (Singapore/0611aTw/2006/FJ807896), 0810bTw (Malaysia/0810bTw/2008/FJ807899) and 0706aTw (Indonesia/0706aTw/2007/FJ807897) were grown on BHK-21 cells [30]. Recombinant baculoviruses (S-WT or control vector) were amplified from infected Sf21 cells [31]. Virus titers were determined using plaque assays or a tissue culture infectious dose 50 assay (TCID_50_). Suramin, purchased from Sigma-Aldrich (catalog # S2671), was freshly dissolved in water to produce a stock solution (50mg/ml, 35mM), which was then stored at -20°C until use.

### Plaque assay and plaque reduction assay

BHK-21 cells were seeded in 6-well plates and incubated at 37°C overnight. In plaque assay, the virus suspension was diluted 10-fold by DMEM containing 2% FBS. To infect BHK-21 cells, we added 0.4 ml viral dilutions to each well. In plaque reduction assay, BHK-21 cells were infected with CHIKV-S27 at a multiplicity of infection (MOI) of 4×10^−4^ in the presence of suramin at indicated concentrations. Following incubation at 37°C for 1 hour, the virus was removed. The infected-cells were covered with 4.5 ml overlay medium containing 1% SeaPlaque agarose (Lonza, catalog # 18104-0807-7) (Suramin at indicated concentrations was added to overlay medium in plaque reduction assay). Cells were then incubated at 37°C for 2 days. Finally, cells were fixed and stained using 1% crystal violet solution to count plaques and determine virus titers, which were presented as plaque-forming units per milliliter (pfu/ml) [32].

### Quantitative real time RT-PCR

Total RNA or viral RNA was isolated using Trizol reagent (Invitrogen, catalog # 15596–026) or the QIAamp Viral RNA Mini Kit (Qiagen, catalog # 52906) in accordance with manufacturer’s instructions. Viral RNA and actin RNA were quantified using the QuantiTect SYBR Green RT-PCR kit (Qiagen, catalog # 204243). Briefly, the primer sequences for CHIKV E1 were as follows: forward, 5’- GTCTGTTCTACACAAGTACAC -3’; reverse, 5’- ACGACACGCATAGCACCAC -3’. Actin was an internal control, and the primer sequences were as follows: forward, 5’- ATTGCCGACAGGATGCAGAA -3’; reverse, 5’- GCTGATCCACATCTGCTGGAA -3’. After 30min at 50°C and 15min at 95°C, forty-five cycles of PCR (one cycle consist of 15 sec at 95°C, 25 sec at 57°C and 10sec at 72°C) were performed using Roche LightCycler 480 System. Melting curve analysis was performed to certify the specificity of PCR products. Relative values were calculated using the ΔΔCt method, and each experiment was performed in triplicate.

### Western blot analysis

Total proteins were dissolved in Laemmli sample buffer and separated onto 10% SDS-PAGE gel. Blotted membranes were incubated using rabbit anti-CHIKV antibodies [31] (1:1000) or mouse anti-actin antibodies (1:1000; Cell Signaling, catalog # 3700). The membranes were then incubated at a 1:2000 dilution with horseradish peroxidase (HRP)-conjugated goat anti-rabbit IgG or anti-mouse IgG at room temperature for 1 hour. HRP was detected on the membrane using a LumiFast Plus Chemiluminescence Detection Kit (T-Pro Biotechnology, catalog # JT96-K002M) in accordance with manufacturer’s protocol. The UVP AutoChemi Image System was used to capture and process images.

### Microneutralization assay

BHK-21, U2OS or MRC-5 cells were seeded in 96-well plates, infected with the CHIKV-S27 strain at an MOI of 0.01 in the presence of suramin at indicated dosages, and incubated for 2 days. The cells were then fixed and stained using 0.1% crystal violet solution at room temperature for 5 minutes, whereupon the stained cells were washed three times [33]. The optical density at 570 nm (OD570) was measured using an Tecan Infinite 200 Pro multiplate reader. All assays were performed at least triplicate. The concentration that achieved 50% of the maximal effect (EC_50_) was calculated using GraphPad Prism Version 5.

### Immunofluorescence assay (IFA)

Infected BHK-21 cells were fixed using an acetone/methanol mixture for 5 minutes and then air-dried for 5 minutes. The cells were subsequently stained with rabbit anti-CHIKV E2 antibodies (1:100) [31] or J2 anti-dsRNA IgG2a monoclonal antibodies (1:100; Scicons, catalog # J2-1406) and incubated at room temperature for 1 hour. After washing with PBS, cells were stained with Alexa Fluor 594-conjugated goat anti-rabbit IgG or anti-mouse IgG (1:500). The cells were then completely covered with DAPI (300nM) for nuclear staining. Images were captured using the red-channel of an inverted fluorescence microscope, to investigate the occurrence of CHIKV infection.

### Binding assay and Entry assay

Binding and entry assays were modified from two methods, plaque reduction assays and focus forming unit reduction assay [34]. The plaque formation was used to evaluate the effects of suramin on binding and entry, based on the fact that holding samples at 4°C can restrict the entry of the virus. BHK-21 cells were infected with the CHIKV-S27 strain at a multiplicity of infection (MOI) of 4×10^−4^ at 4°C (which permits binding but not entry) or at 37°C (which facilitates virus entry / penetration) in the presence of suramin at indicated concentrations before being incubated for 1 hour. The cells were then washed using DMEM to remove the virus and suramin. The infected cells were covered with 4.5 ml of the medium supplemented with 1% agarose prior to incubation at 37°C for 2 days. Finally, the cells were fixed and stained using a 1% solution of crystal violet, and plaques were visualized and counted. The percentage of viruses that succeeded in binding and entry was determined by comparing the number of plaques between viral and control groups. The results were obtained from at least three independent experiments.

### CHIKV 26S mediated insect cell fusion inhibition assay

The CHIKV 26S mediated insect cell fusion inhibition assay was modified from the methods outlined in [35]. Briefly, Sf21 cells were infected with either (1) S-WT recombinant baculovirus for the expression of CHIKV full-length structure protein (26S) or (2) control baculovirus at an MOI of 2 in Sf-900II SFM with 5% FBS. At 1 day post-infection (dpi), the culture medium was replaced with Sf-900II SFM (pH 6.8) supplemented with 2% FBS. At 2 dpi, the infected Sf21 cells were pretreated with suramin or CHIKV neutralization antibodies in the above alkaline medium (pH 6.8) for 1 hour. Cell-cell fusion was subsequently triggered by Sf-900II SFM (pH 5.8) containing 2% FBS and 100μg/ml cholesterol, and treated cells were incubated for 2–3 hrs. Cell fusion was observed using an inverted fluorescence microscope, measured using Image J software, and quantified using the fusion index, as outlined in [35].

### Molecular docking

Docking simulations were performed using PatchDock to generate an ensemble of docked conformations, whereupon scoring functions were used to generate classes based on the dock scores. We then ranked the best conformations [36].

### TCID_50_ assay

BHK-21 cells were seeded on 96-well plates and infected with a series dilution of CHIKV suspensions at 37°C for 4~5 days. TCID_50_ was determined by CHIKV-induced cytopathic effect (CPE), which observed using an inverted microscope.

### Virus stability assay

CHIKV supernatant was administered with suramin at indicated dosages and incubated at 37°C for 8 hours. Viral stability was determined by TCID_50_ assay.

### Cell viability assay

Cell viability profiles were assessed using the WST-1 assay (Roche, catalog # 11644807001) in accordance with manufacturer's protocol. The treated cells were measured absorbance at 440 nm using a Tecan Infinite 200 Pro multiplate reader. All cell viability assays were conducted at least than triplicate, and data for treated cells were normalized using data from untreated cells.

### In vivo toxicity test in zebrafish

The protocol for the zebrafish experiments in this study was approved by the Institutional Animal Care and Use Committee (IACUC) of the College of Medicine, National Taiwan University, and conformed to the criteria outlined in the Guide for the Care and Use of Laboratory Animals of the National Institutes of Health. Toxicity tests were conducted as follows: Stock solutions of suramin were diluted in embryo medium to prepare serial working concentrations. Zebrafish embryos were exposed to suramin solution at the following dosages: 0.7, 7, 70, and 700 uM. Fertilized sphere stage embryos (4–5 h post fertilization, hpf) were kept in 24-well plates at two embryos/well, where each well contained 2 ml of the test solution for 7 days and is updated daily. After exposure, embryos were checked daily for survival, body length, malformation and hatch using a microscope. The hatching rate was expressed as the number of embryos that had hatched, as compared with the control group. The survival rate was expressed as the number of dead embryos as compared with the control group. Morphological anomalies, including chorion with attached debris, delayed development, lack of spontaneous movement at 1 to 7 dpf, pericardial edema, yolk sac edema, bent trunk, tail malformation, and an uninflated swim bladder were observed under stereomicroscopes. The plates were held in an incubator at 28°C photoperiod with a 14/10 h light/dark [37]. All zebrafish after completion of the experiment using 0.5% tricaine (Sigma-Aldrich, catalog # MS-222) sacrificed, and all efforts were made to minimize suffering.

### Statistical analysis

The Student's t test and Kaplan-Meier test were used to analyze data. A p value of <0.05 was considered significant. All statistical analyses were performed using GraphPad prism software.

## Results

### Anti-CHIKV activity of suramin

**Fig 1:**
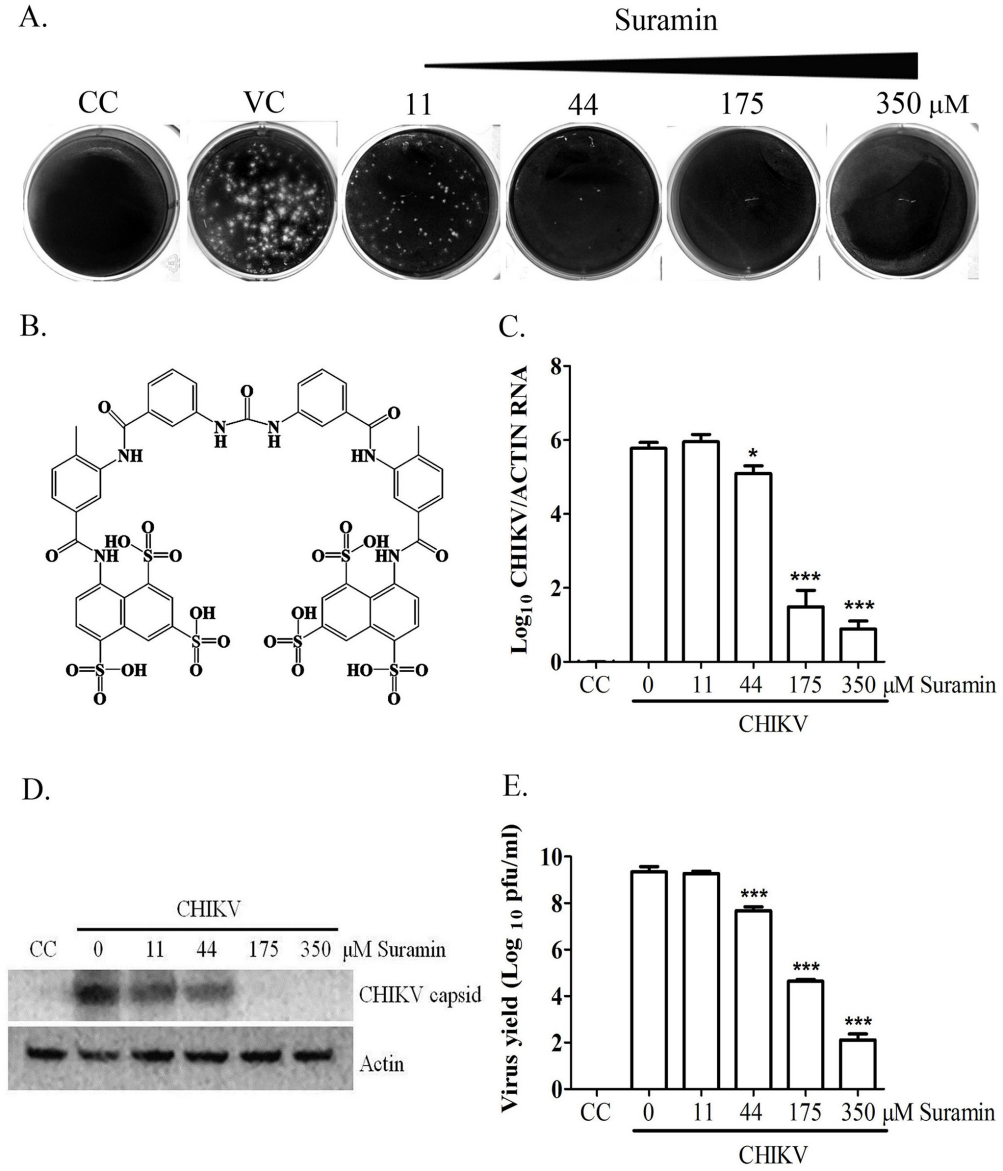
Dose-dependent anti-CHIKV activity of suramin. (A) Plaque reduction assay. (B) Chemical structure of suramin. (C, D, E) BHK-21 cells were infected by CHIKV-S27 strain at an MOI of 0.01 following incubation at 37°C for 1 h in the presence of indicated concentrations of suramin. The infected cells were then incubated in fresh medium with corresponding concentrations of suramin for 24 hours. Total RNA, cell lysate and culture supernatant were harvested. Data of viral RNA (C) were normalized with an internal control of actin, by RT-qPCR. Viral capsid protein (D) and internal control of actin were determined by Western blot analysis. The yield of CHIKV from the supernatant (E) was determined by plaque assay. CC refers to cell control and VC refers to virus control. Each data point was the mean ± SD of three independent experiments. Statistical significance was determined using the t-test compared with the virus control. ***, p< 0.001; **, p< 0.01; *, p< 0.05.

Plaque reduction assays were used to verify the inhibition of suramin via plaque formation in order to determine the effect of suramin on CHIKV infection. BHK-21 cells were commonly used to investigate anti-CHIKV infection [38] because the plaque formation, CPE and cell-to-cell transmission were easily observed. The laboratory-adapted CHIKV-S27 strain was utilized to confirm the anti-viral effects. The results in Fig 1A show that suramin significantly suppressed plaque formation from 44μM to 350μM, which demonstrates the anti-CHIKV activity of suramin. The anti-CHIKV ability of suramin underwent further validation by detecting the production of viral capsid, quantifying the production of viral RNA by RT-qPCR, and determining the yield of infectious progeny virus by plaque assay (Fig 1D and 1E). Our results demonstrate that viral RNA, proteins, and virus yield were significantly reduced by corresponding concentrations of suramin, which confirms the dose-dependent anti-CHIKV activity of suramin.

### Anti-CHIKV activity of suramin on different CHIKV strains

We also investigated the antiviral effects of suramin on CHIKV-S27 strain and three clinically isolated strains: 0611aTw, 0810bTw, and 0706aTw. Microneutralization assay was used to examine the spectrum of anti-CHIKV effect provided by suramin. EC_50_ was used to define the anti-CHIKV effect of suramin and 50% cytotoxicity concentration (CC_50_) was used to evaluate cytotoxicity. Selectivity index (SI = CC_50_/EC_50_) values are presented in Table 1. U2OS and MRC-5 separately belong to human osteosarcoma and human fibroblasts and also utilized to determine the EC_50_ of suramin. The EC_50_ values of CHIKV strains were as follows: 8.8 μM to 28.9 μM (BHK-21 cells), 17.9 μM to 59.6 μM (U2OS cells) and 18.1 μM to 62.1 μM (MRC-5 cells). Those clinical isolates were including an E1-226V mutant strain, Malaysia 0810bTw, which was close to Le Reunion epidemic strain. Suramin revealed broad potential application in clinical CHIKV infection treatment. SI values of CHIKV-S27 on BHK-21, U2OS cells and MRC-5 cells were >32.6, >39.1 and 19.3, respectively. These results demonstrate that suramin also has significant inhibitory effects on clinical isolates including Le Reunion epidemic strain.

**Table 1:** Antiviral and cytotoxic activities of suramin against different CHIKV strains.

Cell lines	EC50^a^ (μM)					
	S27-African prototype	Singapore/0611aTw/2006/FJ807896	Malaysia/0810bTw/2008/FJ807899	Indonesia/0706aTw/2007/FJ807897	CC50^b^ (μM)	SI^c^
BHK-21	21.5±7.1	28.9±6.8	8.8±0.5	21.9±4.8	>700	>32.6
U2OS	17.9±9.5	59.6±11.9	43.8±6.1	36±9.6	>700	>39.1
MRC-5	18.1±4	62.1±5.7	54.1±11.8	54.3±4.7	≒350	19.3

^a^ The EC50s were determined using the microneutralization assay and were presented as means ± SD (n≥3).

^b^ The CC50s were determined using an WST-1 assay and were presented as means ± SD (n≥3).

^c^ The SI (selectively index) represented the ratio of CC50 to EC50 for S27-African prototype.

### Suramin inhibits CHIKV infection in early stages

**Fig 2:**
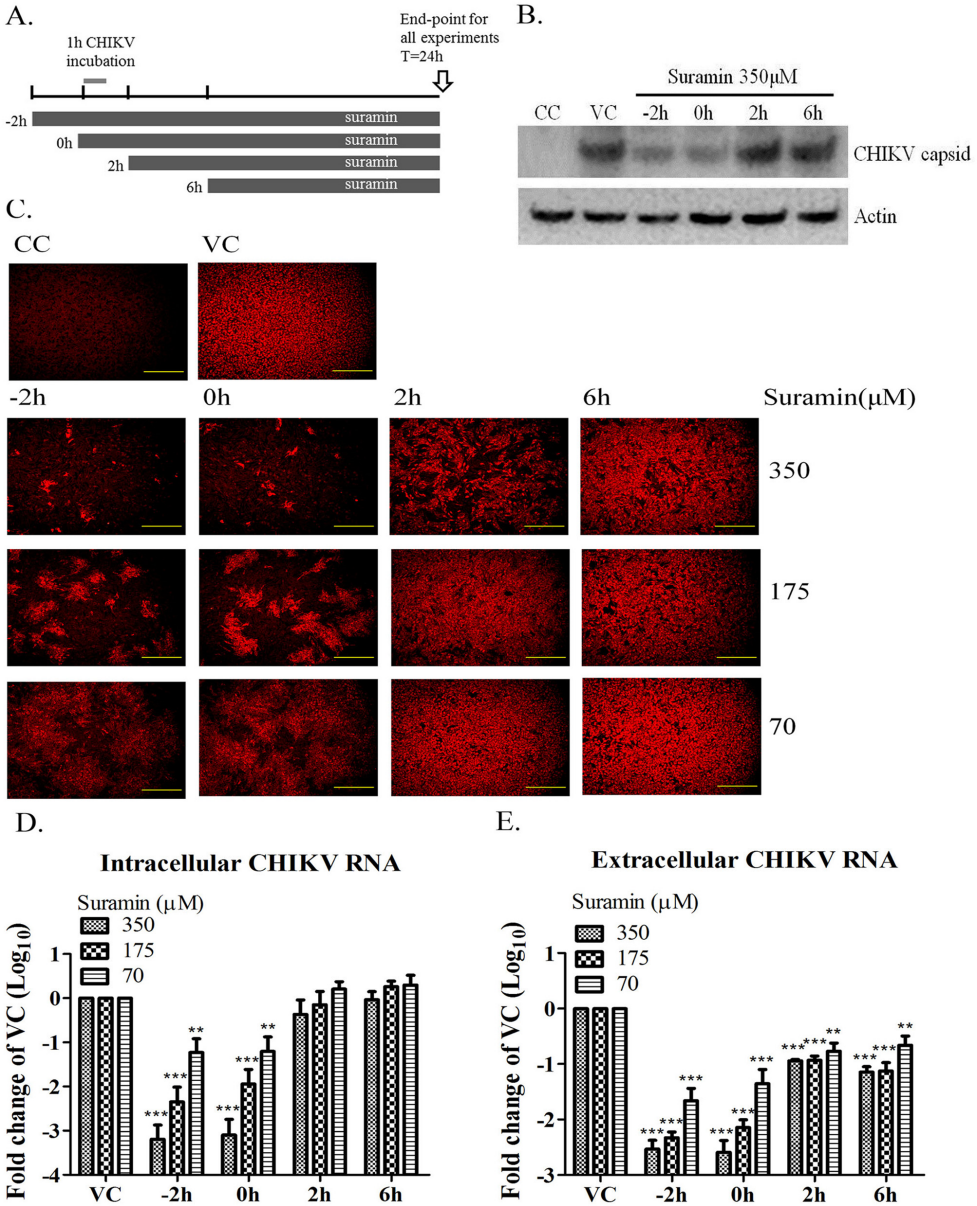
Time-of-addition assay. (A) Time points of suramin administration. BHK-21 cells were infected with CHIKV-S27 strain at an MOI of 0.1 and then incubated for 1 h. Indicated concentrations of suramin were administered at prior to infection (-2h) as well as at 0, 2 and 6h post-infection with CHIKV. All experiments were performed 24 hours after infection. CC refers to cell control and VC refers to virus control. (B) Expression of capsid protein was determined by western blot analysis using an internal control of actin (bottom panel). (C) CHIKV E2 glycoprotein levels were determined by IFA. (D and E) Intracellular and extracellular viral RNA levels were quantified by RT-qPCR performed in triplicate. The fold change was compared with the untreated virus control and presented logarithmically. ***, p< 0.001; **, p< 0.01; *, p< 0.05.

Time-of-addition assay was assessed to identify the stage of CHIKV infection affected by suramin. Suramin was added to BHK-21 cells at different time points from prior to viral infection (-2 hour), viral adsorption (0 hour), in the early (2 hours) and late stages of viral infection (6h) (Fig 2A). The endpoint of this analysis was 24 hours post-infection (hpi). Fig 2B illustrates how suramin treatment significantly inhibited capsid production in the early stage of viral infection (-2 and 0 hour). The same result was observed in E2 staining as well as in intracellular and extracellular CHIKV RNA levels (Fig 2C–2E). We also observed that, under suramin treatment, the size of the CHIKV-infected foci at -2 and 0 hour was restricted, the effects of which were correlated with the dosage of suramin (Fig 2C). These findings indicate that suramin affects CHIKV infection in the early stage of development.

### Suramin disrupts virus binding and fusion by binding with viral glycoproteins

**Fig 3:**
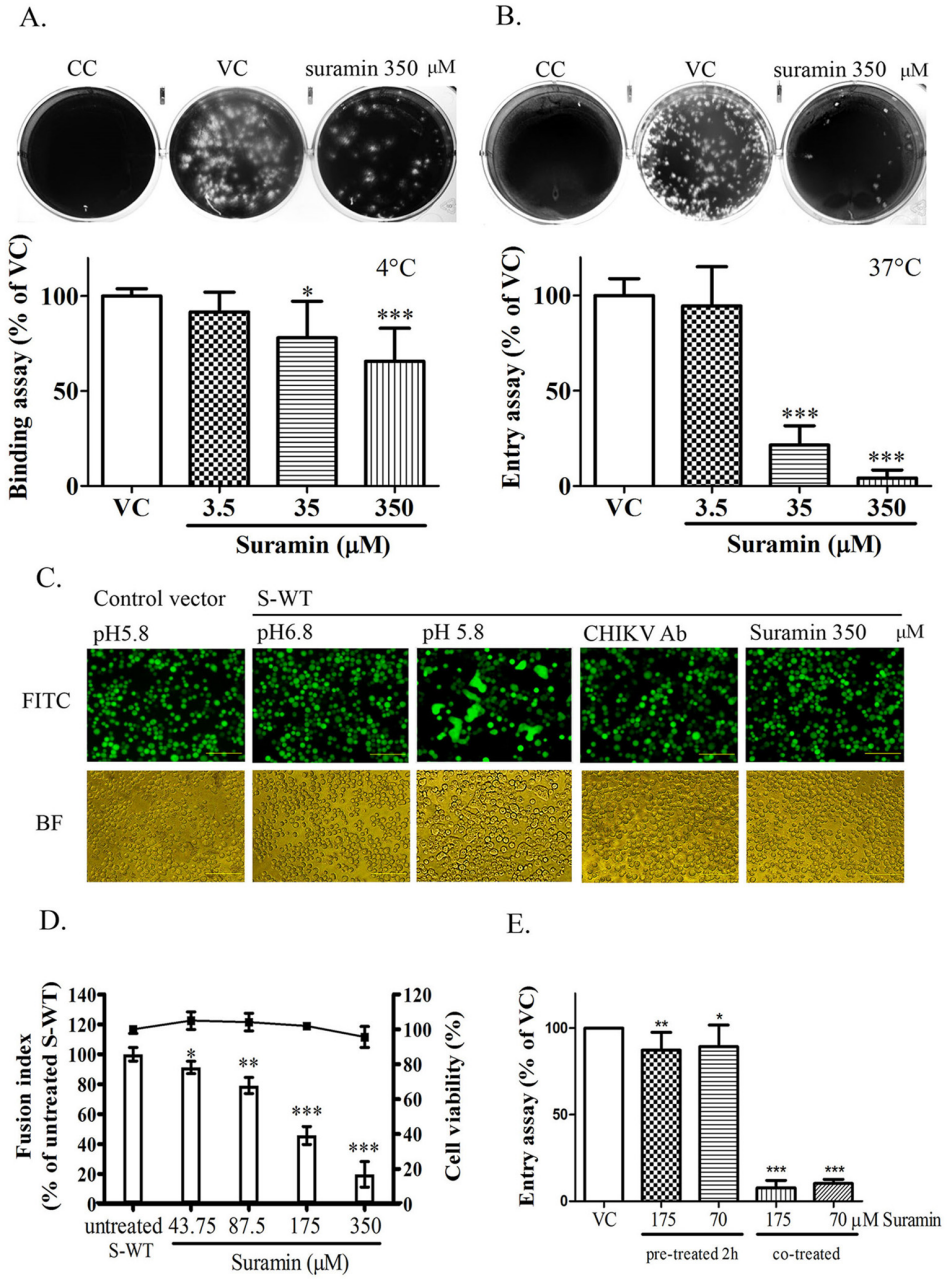
Effect of suramin on CHIKV entry. (A) Binding assay at 4°C. (B) Entry assay at 37°C. The percentage was determined by counting the number of plaque and normalized to that of the virus control group. Each data point is the mean ± SD of at least three independent experiments. ***, p< 0.001; **, p< 0.01; *, p< 0.05. (C) CHIKV 26S-mediated insect cell fusion inhibition assay. Control vector at pH5.8 and recombinant baculovirus S-WT at pH6.8 in parallel serving as a negative control. Cell-cell fusion was induced in recombinant baculovirus S-WT-infected Sf-21 cells by treatment with acid (pH5.8). CHIKV neutralizing antibodies (CHIKV Ab) or suramin (350μM) were used for pre-treatment and co-treatment at pH5.8 in order to assess the effects on inhibiting fusion. Syncytia formation was examined under a fluorescence microscope with an FITC channel (FITC: upper panels) or a bright field channel (BF: lower panels). (D) The percentage of fusion index was calculated and then normalized to the untreated S-WT-infected group. Cell viability of suramin was determined by WST-1 assays. (E) CHIKV MOI = 1, suramin pre-treated 2 hours (BHK-21 cell pre-treated with suramin 2 hours and then washed and replaced to CHIKV (MOI = 1) for 1 hour incubation) and suramin co-treated were analyzed by entry assay. The percentage was determined by counting the number of plaque and normalized to that of the virus control group. Each data point is the mean ± SD of at least three independent experiments. ***, p< 0.001; **, p< 0.01; *, p< 0.05.

Virus-receptor binding and low-pH-triggered membrane fusion reaction are two critical steps associated with CHIKV entry. Lowering the temperature to 4°C was shown to permit virus binding but not entry; therefore, we used temperature to differentiate the effects of virus binding and entry [34]. At concentrations between 35μM and 350μM, suramin was shown to influence virus binding at 4°C (Fig 3A), and significantly reduce virus entry at 37°C (Fig 3B).

We also used the CHIKV 26S mediated insect cell fusion assay to investigate the effects of suramin on virus fusion. CHIKV 26S mediated cell fusion was triggered at pH 5.8; however, this was not observed in the control group and the effect was suppressed by CHIKV neutralizing antibodies, which demonstrates that the fusion assay was CHIKV 26S specific. Suramin was shown to suppress CHIKV 26S mediated cell fusion (Fig 3C) in a dose-dependent manner (Fig 3D). The above evidence suggests that suramin interferes with virus entry via its effects on virus fusion.

Suramin also possess minor effect during virus binding (Fig 3A). To figure out the interaction between suramin and cell receptor, BHK-21 cells was pre-treated with suramin for 2 hours incubation and then replaced to CHIKV for an hour incubation. The results compared to CHIKV only and suramin co-treated groups by entry assay. Co-treated group shown significant inhibition of CHIKV and Pre-treated group also reveal minor inhibition. It indicated that suramin might also minor interact with cell receptor (Fig 3E).

**Fig 4:**
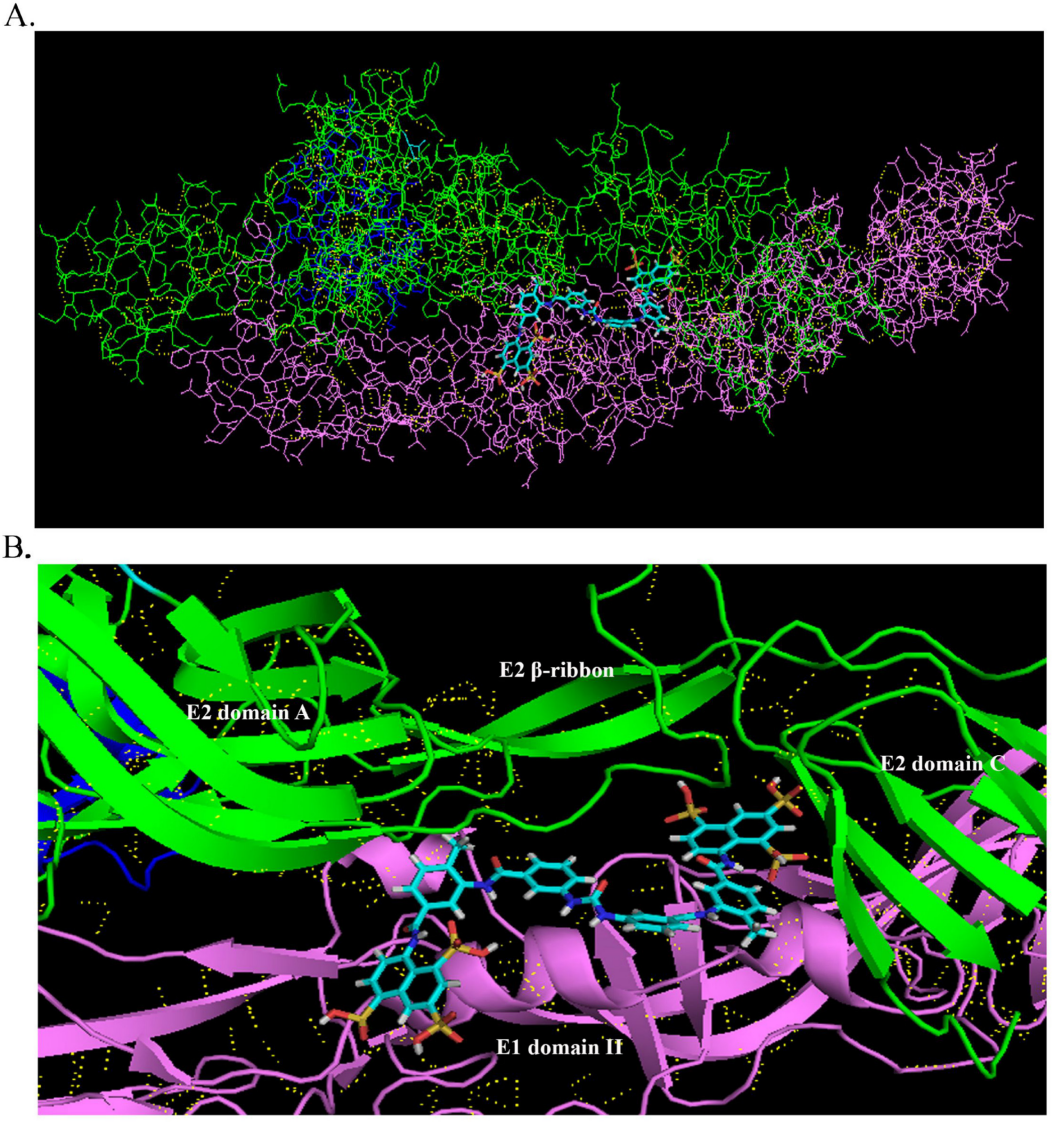
Molecular docking of CHIKV envelope glycoprotein complex and suramin. (A and B) Molecular modeling of the interactions between CHIKV envelope glycoprotein complex (PDB: 3N42) and suramin: Pink lines (E1 domain), Green lines (E2 domain), Blue lines (E3 domain), Yellow lines (hydrogen bond). (B) The highest Patchdock score (9266) was obtained for the model in which suramin embedded within the cavity of the CHIKV envelope glycoprotein complex between E1 and E2.

E2 and E1 glycoproteins regulate virus entry in the early stages of CHIKV infection. E2 was responsible to receptor binding and E1 was shown to form trimers that trigger membrane fusion. This raised the question as to whether suramin interacts with CHIKV glycoproteins. We employed the docking software, PatchDock, to predict the molecular docking of suramin and the CHIKV envelope glycoprotein complex (PDB: 3N42). The highest Patchdock score (9266) was obtained for the model in which suramin embedded within the cavity of the CHIKV envelope glycoprotein complex between E1 domain IIand E2 domain C (Fig 4A and 4B). This implies that suramin may reduce virus fusion and binding by being embedded within this cavity.

### Suramin reduces extracellular transmission by interfering with the release of the virus

**Fig 5:**
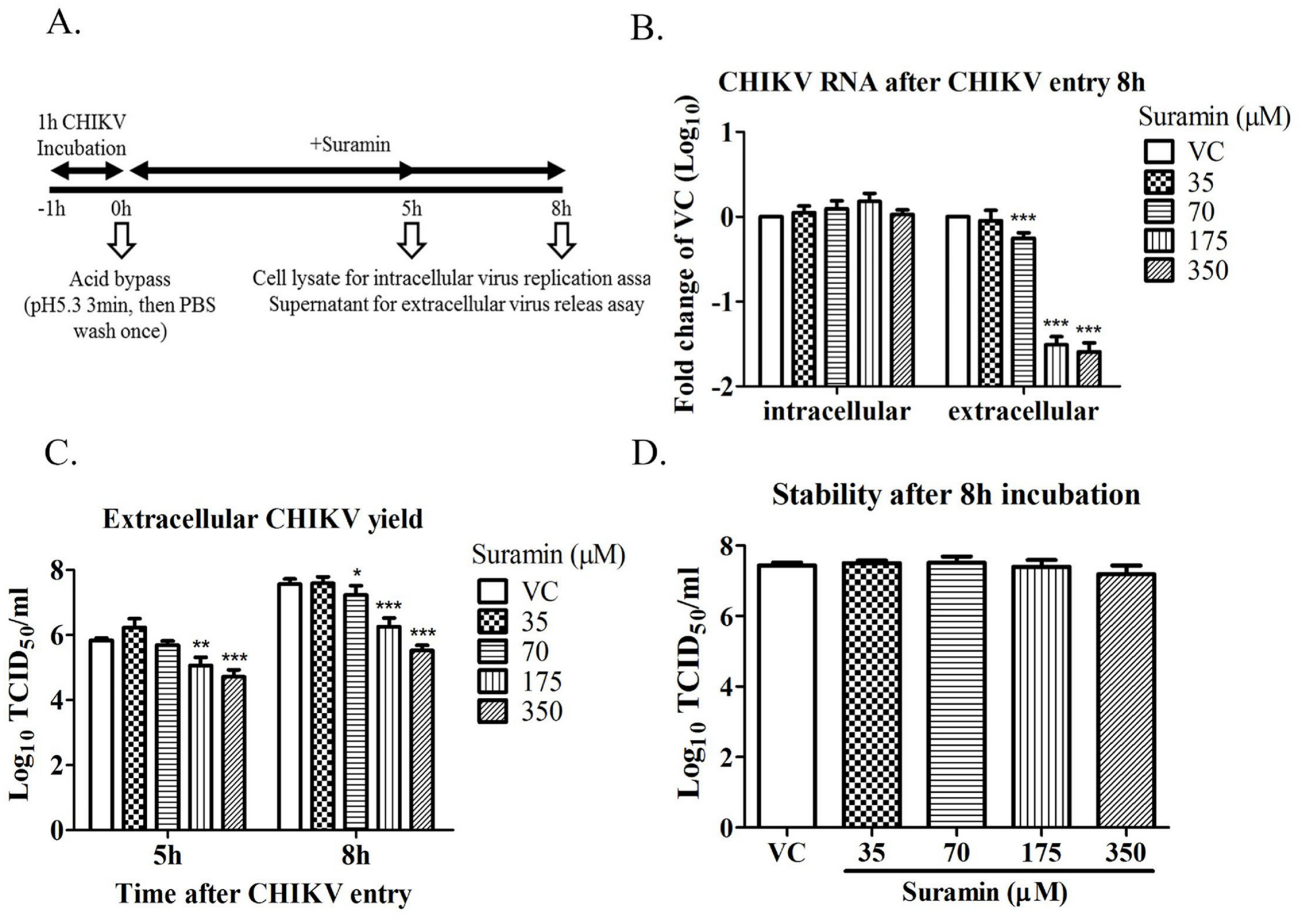
Effect of suramin after CHIKV entry. (A) Timeline of acid-bypass infection. BHK-21 cells infected with CHIKV-S27 strain at an MOI of 1 prior to incubation at 37°C for 1 h. A 3-min DMEM pulse at pH5.3 permitted virus entry into the cytosol. The cells were then washed by PBS once. Afterward, the medium was replaced with 2% FBS DMEM containing indicated concentrations of suramin followed by incubation for 5 hours or 8 hours. The supernatant and cell lysate were collected for RT-qPCR and TCID_50_ assay. (B) The CHIKV RNA change between intracellular and extracellular at 8 hours. The fold change of CHIKV RNA was relative to the untreated virus control (VC) and presented logarithmically. (C) The yield of extracellular CHIKV was determined by TCID_50_ at 5 hours and 8 hours. (D) The virus suspension incubated in the presence of suramin at 37°C for 8 h and virus stability was determined by TCID_50_. Each data point was the mean ± SD of at least three independent experiments. ***, p< 0.001; **, p< 0.01; *, p< 0.05.

The effects of suramin on inhibiting CHIKV entry have been proven; however, it also shown to affect extracellular CHIKV RNA levels following virus entry (Fig 2E). Viral glycoproteins were also shown to participate in the late stage of CHIKV infection. E1/E2 glycoproteins that accumulated on the surface of host cells allowed virus budding and the release of virion particles. Acid bypass infection (pH5.3 for 3 min) allowed CHIKV to bypass the endocytic pathway and enter the cytosol directly through the plasma membrane. Suramin was shown not to influence intracellular CHIKV RNA levels; however, the extracellular CHIKV RNA decreased significantly in a dose-dependent manner (Fig 5B). The yield of CHIKV progeny in the supernatant was also determined according to TCID_50_. Under suramin treatment, extracellular CHIKV titers reduced in a dose-dependent manner from 70 μM to 350 μM either at 5 or 8 hours (Fig 5C). That implies that suramin may reduce extracellular transmission by interfering with the release of the virus. Nonetheless, whether the CHIKV yield decreased in supernatant resulted from suramin disrupt virion stability. An examination of virus stability showed that incubation with suramin for 8 hours did not affect CHIKV stability (Fig 5D). These findings indicate that suramin suppresses the release of the virus but does not have any effect on its stability.

### Effect of suramin on cell-to-cell transmission

**Fig 6:**
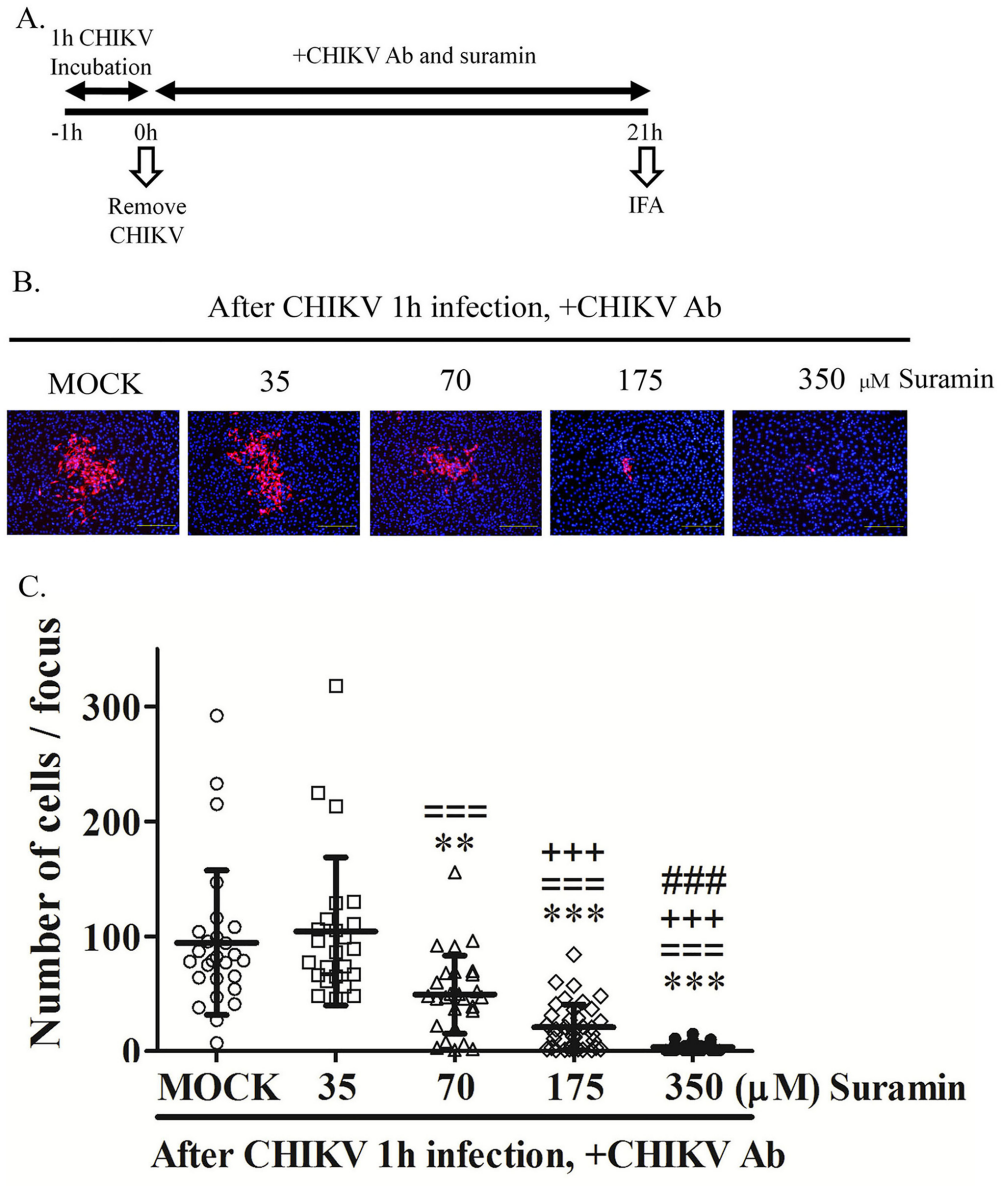
Effect of suramin on cell-to-cell transmission. (A) The timeline illustrates cell-to-cell transmission assays. BHK-21 cells were infected with CHIKV-S27 strain at an MOI of 4×10^−4^ for 1 hour. The medium was them replaced with virus-free medium in the presence of CHIKV neutralizing antibodies (1:100) and suramin at indicated concentrations prior to incubation for 21 hours. Infected cells were stained using dsRNA antibodies (red) for the detection of CHIKV infection and DAPI staining (blue) identified the cell nucleus for CHIKV-infected cell counting (B). (C) Quantification of the number of infected cells (red) per focus (mean ± SD). The significance was calculated using a t-test and shown as * when 35, 70, 175 and 350μM suramin were compared to Mock, as = when 70, 175 and 350μM suramin were compared to 35μM, as + when 175 and 350μM were compared to 70μM, or as # when 350μM was compared to 175μM. The significance is indicated as *, =, +, or # for p<0.05; **, ==, ++, or ## for p<0.01; or ***, ===, +++, or ### for p<0.001.

Time-of-addition experiments showed suramin might restrict the area over which CHIKV spreads (Fig 2C). These results suggest that suramin might affect cell-to-cell transmission. We employed CHIKV neutralizing antibodies to prevent extracellular transmission and the range of CHIKV infection was detected by dsRNA staining. Suramin was shown to have a pronounced effect on reducing the cell-to-cell transmission of CHIKV (Fig 6B). The cell count per focus resulted in Fig 6C suggest that suramin inhibited cell-to-cell transmission in a dose-dependent manner.

## Discussion

Suramin is used for the treatment of trypanosomiasis and onchocerciasis. Previous study reveals that suramin inhibits the replication of Venezuelan equine encephalitis virus by blocking the loading of miRNA onto Ago2 [39]. However, we firstly demonstrated that suramin inhibits CHIKV entry and transmission through binding onto E1/E2 glycoproteins. These findings were firstly confirmed by reductions in plaque formation, viral RNA, proteins, and yield under suramin treatment. Furthermore, suramin was also shown to broadly inhibit 3 clinical isolates including a 226V mutant strain, Malaysia 0810bTw, with EC_50_ values ranging from 8.8 μM to 62.1 μM. Cell viability assay revealed that the CC_50_ of suramin at 48 hours was more than 700 μM in BHK-21 cells and U2OS cells and about 350 μM in MRC-5 cells (S1A Fig). Suramin was shown to be non-toxic to zebrafish following treatment with 700 μM. After exposure, the embryos were no significant variation for survival, body length, malformation and hatch. (S1B Fig). Pharmacokinetics data from previous studies revealed that serum concentrations of suramin in humans exceeded 70 μM (equal to 100μg/ml)[40]. This concentration is well within the anti-viral active concentration range (EC_50_ of 8.8 to 62.1 μM), which is non-toxic in humans.

Suramin was shown to inhibit the early stage of enterovirus 71 infection by blocking binding on the surface of virion [28]. In this study, early treatment with suramin (-2 and 0 hour) also had a pronounced effect on the entry stages of CHIKV infection. However, we found that suramin affected on virus fusion better than virus binding. CHIKV fusion was associated with irreversible E1 trimer formation. Previous study indicated that 7 novel binding sites between E1 and E2 glycoproteins have been revealed for design of inhibitors that could alter the function of the envelope proteins [41,42]. Molecular docking predicts that suramin may embed within the cavity between E1 domain II and E2 domain C. These results help to elucidate the mechanism by which suramin reduces CHIKV fusion and partial binding. Suramin also possessed minor effect on cell receptor. Heparin and heparan sulfate had been reported involved CHIKV attachment [43,44]. Suramin was an analogue of heparin [45,46], so might interact with receptor.

In time-of-addition assays, Suramin also revealed minor effect in the later stages of CHIKV infection. By interfering with the release of CHIKV, suramin was shown to repress extracellular transmission; however, the mechanism by which this occurs has yet to be elucidated. No effects were observed with regard to the stability of CHIKV progeny after incubation with suramin for 8 hr. Alphavirus E1 and E2 proteins are crucial to virus entry and assembly, both of which are associated with protein reorganization [47]. Specific E1 antibodies significantly decrease the release of CHIKV, which implies that E1 may be involved in virus release [48]. Thus, the interaction of suramin and E1/E2 glycoproteins may also interfere with the process of viral release. Previous study indicated that suramin is an inhibitor of norovirus RNA polymerase capable of blocking virus replication [27]. However, following the administration of suramin, we did not observe any difference in intracellular CHIKV RNA after CHIKV entry. Suramin bears strongly hydrophilic polysulfonate groups, which might limit accessibility across cellular membranes [49].

Many viruses including HIV, HSV, HCV, measles, and CHIKV [50–53] spread through cell-cell transmission and viral glycoproteins have been shown to play a critical role regulating cell-to-cell transmission [18]. Suramin significantly restricted the spreading of CHIKV from cell to cell in a dose-dependent manner. This provides further evidence that the interaction between suramin and CHIKV glycoproteins may affect cell-to-cell transmission.

In summary, this study demonstrated that suramin inhibits CHIKV infection by interfering with viral entry (binding/fusion) as well as extracellular and cell-to-cell transmission. Molecular docking indicates suramin would embed within the cavity of the E1/E2 heterodimer, which provides a possible interpretation for suramin targeting ingress and egress of CHIKV infection. This makes suramin a new candidate drug to deal with CHIKV infection.

## Supporting Information

S1 Fig Toxicity of suramin. (A) Cytotoxicity assay. BHK-21, U2OS and MRC-5 cells were treated with suramin at indicated concentrations. After incubation for 24 h and 48h, cell viability was determined using WST-1 assay and was normalized with cell control. ***, p< 0.001; **, p< 0.01; *, p< 0.05. (B) Toxicity assay on zebrafish. Kaplan-Meier plot of survival in larvae fish exposed to suramin (700uM to 0.7uM) for 7 days. n≥ 70 fish per treatment.
(TIF)

## References

[1] Higgs S (2006) The 2005–2006 Chikungunya epidemic in the Indian Ocean. Vector Borne Zoonotic Dis 6: 115–116. 16796508 10.1089/vbz.2006.6.115

[2] Renault P, Solet JL, Sissoko D, Balleydier E, Larrieu S, Filleul L, et al (2007) A major epidemic of chikungunya virus infection on Reunion Island, France, 2005–2006. Am J Trop Med Hyg 77: 727–731. 17978079

[3] Khan K, Bogoch I, Brownstein JS, Miniota J, Nicolucci A, Hu W, et al (2014) Assessing the origin of and potential for international spread of chikungunya virus from the Caribbean. PLoS Curr 6. 10.1371/currents.outbreaks.2134a0a7bf37fd8d388181539fea2da5 PMC4055609 24944846

[4] Fischer M, Staples JE, Arboviral Diseases Branch NCfE, Zoonotic Infectious Diseases CDC, et al (2014) Notes from the field: chikungunya virus spreads in the Americas—Caribbean and South America, 2013–2014. MMWR Morb Mortal Wkly Rep 63: 500–501. 24898168 PMC5779358

[5] Morrison TE (2014) Reemergence of chikungunya virus. J Virol 88: 11644–11647. 10.1128/JVI.01432-14 25078691 PMC4178719

[6] Burt FJ, Rolph MS, Rulli NE, Mahalingam S, Heise MT. (2012) Chikungunya: a re-emerging virus. Lancet 379: 662–671. 10.1016/S0140-6736(11)60281-X 22100854

[7] Schwartz O, Albert ML (2010) Biology and pathogenesis of chikungunya virus. Nat Rev Microbiol 8: 491–500. 10.1038/nrmicro2368 20551973

[8] Couderc T, Chretien F, Schilte C, Disson O, Brigitte M, Guivel-Benhassine F, et al (2008) A mouse model for Chikungunya: young age and inefficient type-I interferon signaling are risk factors for severe disease. PLoS Pathog 4: e29 10.1371/journal.ppat.0040029 18282093 PMC2242832

[9] Noret M, Herrero L, Rulli N, Rolph M, Smith PN, Li RW, et al (2012) Interleukin 6, RANKL, and osteoprotegerin expression by chikungunya virus-infected human osteoblasts. J Infect Dis 206: 455–457: 457–459. 10.1093/infdis/jis368 22634878

[10] Ozden S, Huerre M, Riviere JP, Coffey LL, Afonso PV, Mouly V, et al (2007) Human muscle satellite cells as targets of Chikungunya virus infection. PLoS One 2: e527 17565380 10.1371/journal.pone.0000527 PMC1885285

[11] Her Z, Malleret B, Chan M, Ong EK, Wong SC, Kwek DJ, et al (2010) Active infection of human blood monocytes by Chikungunya virus triggers an innate immune response. J Immunol 184: 5903–5913. 10.4049/jimmunol.0904181 20404274

[12] Labadie K, Larcher T, Joubert C, Mannioui A, Delache B, Brochard P, et al (2010) Chikungunya disease in nonhuman primates involves long-term viral persistence in macrophages. J Clin Invest 120: 894–906. 10.1172/JCI40104 20179353 PMC2827953

[13] Rochlin I, Ninivaggi DV, Hutchinson ML, Farajollahi A (2013) Climate change and range expansion of the Asian tiger mosquito (Aedes albopictus) in Northeastern USA: implications for public health practitioners. PLoS One 8: e60874 10.1371/journal.pone.0060874 23565282 PMC3614918

[14] Voss JE, Vaney MC, Duquerroy S, Vonrhein C, Girard-Blanc C, Crublet E, et al (2010) Glycoprotein organization of Chikungunya virus particles revealed by X-ray crystallography. Nature 468: 709–712. 10.1038/nature09555 21124458

[15] Hahon N, Zimmerman WD (1970) Chikungunya virus infection of cell monolayers by cell-to-cell and extracellular transmission. Appl Microbiol 19: 389–391. 4908535 10.1128/am.19.2.389-391.1970 PMC376692

[16] Soonsawad P, Xing L, Milla E, Espinoza JM, Kawano M, Marko M, et al (2010) Structural evidence of glycoprotein assembly in cellular membrane compartments prior to Alphavirus budding. J Virol 84: 11145–11151. 10.1128/JVI.00036-10 20739526 PMC2953181

[17] Garoff H, Sjoberg M, Cheng RH. (2004) Budding of alphaviruses. Virus Res 106: 103–116. 15567491 10.1016/j.virusres.2004.08.008

[18] Lee CY, Kam YW, Fric J, Malleret B, Koh EG, Prakash C, et al (2011) Chikungunya virus neutralization antigens and direct cell-to-cell transmission are revealed by human antibody-escape mutants. PLoS Pathog 7: e1002390 10.1371/journal.ppat.1002390 22144891 PMC3228792

[19] Mitsuya H, Popovic M, Yarchoan R, Matsushita S, Gallo RC, Broder S. (1984) Suramin protection of T cells in vitro against infectivity and cytopathic effect of HTLV-III. Science 226: 172–174. 6091268 10.1126/science.6091268

[20] Yahi N, Sabatier JM, Nickel P, Mabrouk K, Gonzalez-Scarano F, Fantini J. (1994) Suramin inhibits binding of the V3 region of HIV-1 envelope glycoprotein gp120 to galactosylceramide, the receptor for HIV-1 gp120 on human colon epithelial cells. J Biol Chem 269: 24349–24353. 7929093

[21] Aguilar JS, Rice M, Wagner EK. (1999) The polysulfonated compound suramin blocks adsorption and lateral difusion of herpes simplex virus type-1 in vero cells. Virology 258: 141–151. 10329576 10.1006/viro.1999.9723

[22] Kessler HA, Pottage JC Jr., Trenholme GM, Benson CA, Levin S. (1986) Effects of suramin on in vitro HBsAg production by PLC/PRF/5 cells and hepatitis B virus DNA polymerase activity. AIDS Res 2: 63–72. 2424468 10.1089/aid.1.1986.2.63

[23] Garson JA, Lubach D, Passas J, Whitby K, Grant PR. (1999) Suramin blocks hepatitis C binding to human hepatoma cells in vitro. J Med Virol 57: 238–242. 10022794

[24] Basavannacharya C, Vasudevan SG (2014) Suramin inhibits helicase activity of NS3 protein of dengue virus in a fluorescence-based high throughput assay format. Biochem Biophys Res Commun. 453: 539–44. 10.1016/j.bbrc.2014.09.113 25281902

[25] Xu K, Ren H, Zhu J, Yang Y, Liao F (2003) Suramin inhibits the in vitro expression of encephalitis B virus proteins NS3 and E. J Huazhong Univ Sci Technolog Med Sci 23: 375–379. 15015640 10.1007/BF02829422

[26] Jiao L, Ouyang S, Liang M, Niu F, Shaw N, Wu W, et al (2013) Structure of severe fever with thrombocytopenia syndrome virus nucleocapsid protein in complex with suramin reveals therapeutic potential. J Virol 87: 6829–6839. 10.1128/JVI.00672-13 23576501 PMC3676114

[27] Croci R, Pezzullo M, Tarantino D, Milani M, Tsay SC, Sureshbabu R, et al (2014) Structural bases of norovirus RNA dependent RNA polymerase inhibition by novel suramin-related compounds. PLoS One 9: e91765 10.1371/journal.pone.0091765 24622391 PMC3951423

[28] Wang Y, Qing J, Sun Y, Rao Z. (2014) Suramin inhibits EV71 infection. Antiviral Res 103: 1–6. 10.1016/j.antiviral.2013.12.008 24374150

[29] Ellenbecker M, Lanchy JM, Lodmell JS. (2014) Inhibition of Rift Valley fever virus replication and perturbation of nucleocapsid-RNA interactions by suramin. Antimicrob Agents Chemother. 10.1128/AAC.03595-14 PMC4249559 25267680

[30] Huang JH, Yang CF, Su CL, Chang SF, Cheng CH, Yu SK, et al (2009) Imported chikungunya virus strains, Taiwan, 2006–2009. Emerg Infect Dis 15: 1854–1856. 10.3201/eid1511.090398 19891886 PMC2857229

[31] Kuo SC, Chen YJ, Wang YM, Kuo MD, Jinn TR, Chen WS, et al (2011) Cell-based analysis of Chikungunya virus membrane fusion using baculovirus-expression vectors. J Virol Methods 175: 206–215. 10.1016/j.jviromet.2011.05.015 21619896

[32] Shih SR, Horng JT, Poon LL, Chen TC, Yeh JY, Hsieh HP, et al (2010) BPR2-D2 targeting viral ribonucleoprotein complex-associated function inhibits oseltamivir-resistant influenza viruses. J Antimicrob Chemother 65: 63–71. 10.1093/jac/dkp393 19892833

[33] Cruz DJ, Bonotto RM, Gomes RG, da Silva CT, Taniguchi JB, No JH, et al (2013) Identification of novel compounds inhibiting chikungunya virus-induced cell death by high throughput screening of a kinase inhibitor library. PLoS Negl Trop Dis 7: e2471 10.1371/journal.pntd.0002471 24205414 PMC3814572

[34] Moghaddam E, Teoh BT, Sam SS, Lani R, Hassandarvish P, Chik Z, et al (2014) Baicalin, a metabolite of baicalein with antiviral activity against dengue virus. Sci Rep 4: 5452 10.1038/srep05452 24965553 PMC4071309

[35] Kuo SC, Chen YJ, Wang YM, Tsui PY, Kuo MD, Wu TY, et al (2012) Cell-based analysis of Chikungunya virus E1 protein in membrane fusion. J Biomed Sci 19: 44 10.1186/1423-0127-19-44 22520648 PMC3384457

[36] Schneidman-Duhovny D, Inbar Y, Nussinov R, Wolfson HJ. (2005) PatchDock and SymmDock: servers for rigid and symmetric docking. Nucleic Acids Res 33: W363–367. 15980490 10.1093/nar/gki481 PMC1160241

[37] Lammer E, Carr GJ, Wendler K, Rawlings JM, Belanger SE. (2009) Is the fish embryo toxicity test (FET) with the zebrafish (Danio rerio) a potential alternative for the fish acute toxicity test? Comp Biochem Physiol C Toxicol Pharmacol 149: 196–209. 10.1016/j.cbpc.2008.11.006 19095081

[38] Kaur P, Thiruchelvan M, Lee RC, Chen H, Chen KC, Ng ML, et al (2013) Inhibition of chikungunya virus replication by harringtonine, a novel antiviral that suppresses viral protein expression. Antimicrob Agents Chemother 57: 155–167. 10.1128/AAC.01467-12 23275491 PMC3535938

[39] Madsen C, Hooper I, Lundberg L, Shafagati N, Johnson A, Senina S, et al (2014) Small molecule inhibitors of Ago2 decrease Venezuelan equine encephalitis virus replication. Antiviral Res 112: 26–37. 10.1016/j.antiviral.2014.10.002 25448087

[40] Chijioke CP, Umeh RE, Mbah AU, Nwonu P, Fleckenstein LL. (1998) Clinical pharmacokinetics of suramin in patients with onchocerciasis. Eur J Clin Pharmacol 54: 249–251. 9681668 10.1007/s002280050454

[41] Rashad AA, Keller PA (2013) Structure based design towards the identification of novel binding sites and inhibitors for the chikungunya virus envelope proteins. J Mol Graph Model 44: 241–252. 10.1016/j.jmgm.2013.07.001 23911992

[42] Rashad AA, Mahalingam S, Keller PA. (2014) Chikungunya virus: emerging targets and new opportunities for medicinal chemistry. J Med Chem 57: 1147–1166. 10.1021/jm400460d 24079775

[43] Gardner CL, Hritz J, Sun C, Vanlandingham DL, Song TY, Ghedin E, et al (2014) Deliberate attenuation of chikungunya virus by adaptation to heparan sulfate-dependent infectivity: a model for rational arboviral vaccine design. PLoS Negl Trop Dis 8: e2719 10.1371/journal.pntd.0002719 24587470 PMC3930508

[44] Silva LA, Khomandiak S, Ashbrook AW, Weller R, Heise MT, Morrison TE, et al (2014) A single-amino-acid polymorphism in Chikungunya virus E2 glycoprotein influences glycosaminoglycan utilization. J Virol 88: 2385–2397. 10.1128/JVI.03116-13 24371059 PMC3958064

[45] Tan CW, Poh CL, Sam IC, Chan YF. (2013) Enterovirus 71 uses cell surface heparan sulfate glycosaminoglycan as an attachment receptor. J Virol 87: 611–620. 10.1128/JVI.02226-12 23097443 PMC3536405

[46] Basu A, Beyene A, Meyer K, Ray R. (2004) The hypervariable region 1 of the E2 glycoprotein of hepatitis C virus binds to glycosaminoglycans, but this binding does not lead to infection in a pseudotype system. J Virol 78: 4478–4486. 15078928 10.1128/JVI.78.9.4478-4486.2004 PMC387685

[47] Sjoberg M, Garoff H (2003) Interactions between the transmembrane segments of the alphavirus E1 and E2 proteins play a role in virus budding and fusion. J Virol 77: 3441–3450. 12610119 10.1128/JVI.77.6.3441-3450.2003 PMC149539

[48] Masrinoul P, Puiprom O, Tanaka A, Kuwahara M, Chaichana P, Ikuta K, et al (2014) Monoclonal antibody targeting chikungunya virus envelope 1 protein inhibits virus release. Virology 464–465: 111–117. 10.1016/j.virol.2014.05.038 25063884

[49] Mastrangelo E, Mazzitelli S, Fabbri J, Rohayem J, Ruokolainen J, Nykänen A, et al (2014) Delivery of Suramin as an Antiviral Agent through Liposomal Systems. ChemMedChem 9: 933–939. 10.1002/cmdc.201300563 24616282

[50] Dingwell KS, Brunetti CR, Hendricks RL, Tang Q, Tang M, Rainbow AJ, et al (1994) Herpes simplex virus glycoproteins E and I facilitate cell-to-cell spread in vivo and across junctions of cultured cells. J Virol 68: 834–845. 8289387 10.1128/jvi.68.2.834-845.1994 PMC236520

[51] Monel B, Beaumont E, Vendrame D, Schwartz O, Brand D, Mammano F. (2012) HIV cell-to-cell transmission requires the production of infectious virus particles and does not proceed through env-mediated fusion pores. J Virol 86: 3924–3933. 10.1128/JVI.06478-11 22258237 PMC3302491

[52] Al Olaby RR, Cocquerel L, Zemla A, Saas L, Dubuisson J, Vielmetter J, et al (2014) Identification of a novel drug lead that inhibits HCV infection and cell-to-cell transmission by targeting the HCV E2 glycoprotein. PLoS One 9: e111333 10.1371/journal.pone.0111333 25357246 PMC4214736

[53] Brindley MA, Chaudhury S, Plemper RK (2015) Measles Virus Glycoprotein Complexes Preassemble Intracellularly and Relax during Transport to the Cell Surface in Preparation for Fusion. J Virol 89: 1230–1241. 10.1128/JVI.02754-14 25392208 PMC4300639

