# Supplementary figures and images for: Suramin Inhibits Chikungunya Virus Entry and Transmission
Source: PLoS One. 2015 Jul 24;10(7):e0133511. doi: 10.1371/journal.pone.0133511 (PMC4514758; doi:10.1371/journal.pone.0133511)

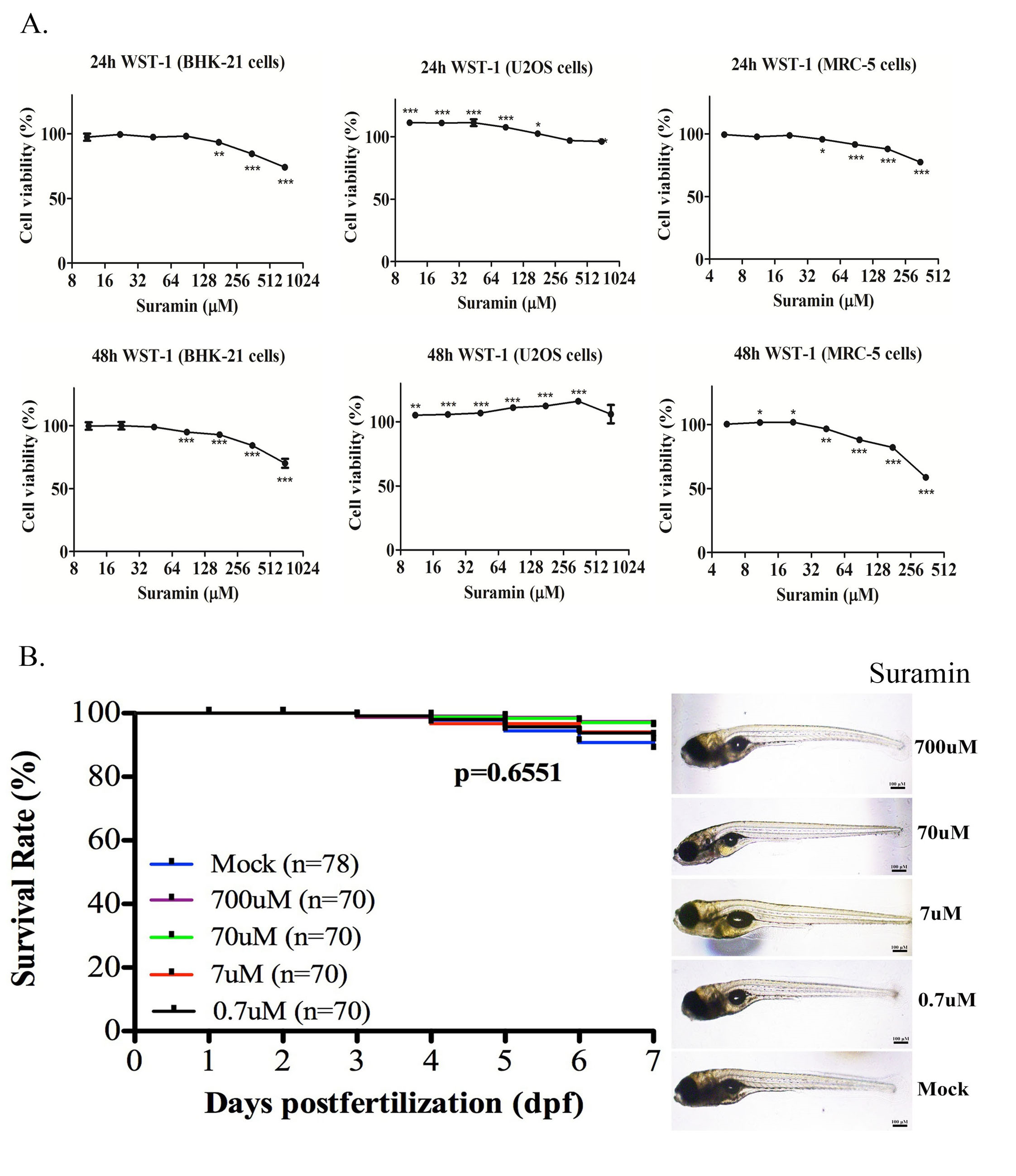

Supplement: S1 Fig — (A) Cytotoxicity assay. BHK-21, U2OS and MRC-5 cells were treated with suramin at indicated concentrations. After incubation for 24 h and 48h, cell viability was determined using WST-1 assay and was normalized with cell control. ***, p< 0.001; **, p< 0.01; *, p< 0.05. (B) Toxicity assay on zebrafish. Kaplan-Meier plot of survival in larvae fish exposed to suramin (700uM to 0.7uM) for 7 days. n≥ 70 fish per treatment. (TIF) [file pone.0133511.s001.tif]
